# Inhibition of CARM1 suppresses proliferation of multiple myeloma cells through activation of p53 signaling pathway

**DOI:** 10.1007/s11033-023-08645-5

**Published:** 2023-07-21

**Authors:** Lan Yang, Le Ma, Qiang Gong, JiePing Chen, Qilin Huang

**Affiliations:** 1 Medical College of Guizhou University, Guiyang City, 550025 China; 2 Department of Hematology, Southwest Hospital, Third Military Medical University (Army Medical University), Gaotanyan Road Street, Shapingba District, 400038 China; 3 Department of Neurosurgery, Guiqian International General Hospital, Changpo Road, Wudang District, Guiyang City, 550000 China; 4 Institute of Rocket Force Medicine, State Key Laboratory of Trauma, Burns and Combined Injury, Army Medical University), Third Military Medical University, Chongqing, 400038 China

**Keywords:** Multiple myeloma, CARM1, Cell proliferation, p53

## Abstract

**Background:**

Multiple myeloma (MM) is a malignant proliferative disease of plasma cells, the incidence of which is increasing every year and remains incurable. The enzyme co-activator-associated arginine methyltransferase 1 (CARM1) is highly expressed in a variety of cancers, such as Hodgkin's lymphoma and acute myeloid leukemia, and CARM1 is closely associated with tumor cell proliferation. However, the role of CARM1 in MM has not been elucidated.

**Methods and results:**

In this study, we found that CARM1 is overexpressed in MM and closely associated with poor prognosis in MM. CCK-8 and colony formation assays showed that the proliferation of MM cell lines was downregulated when CARM1 expression was knockdown by specific shRNA. Knockdown of CARM1 reduced the proportion of MM cell lines in the S phase and increased the proportion in G0/G1 phase. RNA-seq analysis of the CARM1-KD cell line revealed that it was closely associated with apoptosis and activated the p53 pathway. CCK-8 and apoptosis results showed that CARM1 knockdown made MM cells more sensitive to standard-of-care drugs.

**Conclusion:**

This study provides an experimental basis for elucidating the pathogenesis of multiple myeloma and searching for potential therapeutic targets.

## Introduction

Multiple myeloma (MM) is a clonal malignancy that accumulates plasma cells in the bone marrow (BM). This disease causes bone lesions, renal failure, and immunodeficiency [1]. Among hematologic tumors, MM has the second highest incidence rate, after lymphoma [2]. The prevalence has increased dramatically in recent years, with a higher prevalence in middle-aged and older men than in women. It has been estimated that the median survival time for patients with MM is about 6 years [3]. Those with MM would suffer a serious reduction in quality of life and long-term survival. Current treatments include chemotherapy, bone marrow transplantation, cell therapy, and new targeted drugs [4, 5]. Despite the significant improvement in disease response rates, there are still some patients who do not benefit from current treatment and achieve long-term survival. Therefore, it is of great clinical significance to further study the pathogenesis of MM and find new potential targets for the treatment of MM.

CARM1 (The enzyme co-activator-associated arginine methyltransferase 1) is an epigenetic modifying enzyme, also known as protein arginine methyltransferase 4 (PRMT4), which belongs to type I arginine methyltransferase and is asymmetrically dimethylated to histone arginine residues. It has been reported in the literature that CARM1 is overexpressed in a variety of cancer types (e.g., breast [6, 7], ovarian [8, 9], pancreatic [10], liver [11], and hematologic tumors [12, 13]). In addition, CARM1 is also associated with proliferation and metastasis. Shijie Li et al. reported that the overexpression of CARM1 is associated with the Enneking stage of OS (Osteosarcoma) and the knockdown of CARM1 expression decreased proliferation through the pGSK3 β/β-Catenin/cyclinD1 Signaling Pathway and affected the cell cycle in OS cell lines [14]. Interestingly, it has been reported that loss of CARM1 has little effect on normal hematopoietic function, but knockdown of CARM1 impairs cell cycle progression, promotes myeloid differentiation, and ultimately induces apoptosis [15]. Some studies have confirmed the involvement of CARM1 in the pathogenesis of MM, and its selective inhibitors (e.g., EZM2302, TP064) have been shown to inhibit the tumor cell activity of MM [16, 17]. GEO analysis suggests that high CARM1 expression is closely associated with poor prognosis of MM, but the specific mechanism of CARM1 involvement in MM pathogenesis has not been reported.

To explore the role of CARM1 in the pathogenesis of MM, we examined the expression level of CARM1 in bone marrow specimens from MM patients and also investigated how CARM1 is involved in the pathogenesis of MM by affecting cell proliferation at the cellular level. RNA-Seq analysis of CARM1-KD cell lines revealed that it was closely associated with apoptosis and that the p53 signaling pathway was significantly enriched. Our study suggests that CARM1 may promote MM cell proliferation by regulating p53, and its expression level is closely related to the prognosis of MM while providing a potential target for MM treatment.

## Materials and methods

### Antibodies and reagents

The antibodies were used as following, CARM1(3379S, Cell Signaling Technology), β-actin(3700S, Cell Signaling Technology), p53(2527 T, Cell Signaling Technology), p21(2947 T, Cell Signaling Technology), PARP(9542 T, Cell Signaling Technology), cleaved-caspase-3 (9661 T, Cell Signaling Technology), BAF155(11956S, Cell Signaling Technology), BAF155me2a(94962S, Cell Signaling Technology),CDK4(12790 T, Cell Signaling Technology), CDK6(13331 T, Cell Signaling Technology), HRP-conjugated secondary antibodies were anti-mouse and anti-rabbit IgG (7076 and 7074, Cell Signaling Technology). CCK-8 Cell Proliferation and Cytotoxicity Assay Kit (CA1210, Solarbio), EdU Alexa Fluor™ 647 Flow Cytometry Assay Kit (C10634, Invitrogen™ Click-iT™ Plus), APC Annexin V Apoptosis Detection Kit (640,932, Biolegend), RNAiso Plus (9108, Takara), PrimeScript RT reagent Kit with gDNA Eraser (RR047A, Takara), SYBR Green master Mix (208,054, Qiagen), Bortezomib (HY-10227, MCE).

### Cell lines and culture

Human MM cell lines, KMS-11, RPMI-8226, NCI-H929, U266B1, AMO1, L363, OPM-2, were purchased by MeisenCTCC. The cells were cultured in RPMI-1640 and supplemented with 10% fetal bovine serum (FBS) (FB25015, Clark Bioscience), penicillin and streptomycin solution (100 µg/mL, 15,140,122, Gibco) under the condition of 37 °C in a humidified atmosphere of 5% CO_2_.

### Immunohistochemistry analysis (IHC)

The paraffin-embedded patients’ tissues were dewaxed and rehydrated and incubated with human CARM1 antibody (1: 200, ab84370, abcam) at 4 °C overnight. Then, the slides were sequentially incubated with secondary antibody at 37 °C for 1 h and used DAB to visualize positive staining.

CARM1 expression in MM patients’ tissues was evaluated by the percentage of positive staining cells. The intensity was graded as follows: 0, no signal; 1, weak (light yellow); 2, moderate (brown); and 3, strong staining (sepia). The percentage of positive cells was evaluated quantitatively and scored as 0 (< 5% positive tumor cells), 1 (5–25% positive tumor cells), 2 (26–50% positive tumor cells), 3 (51–75% positive tumor cells) and 4 (> 75% positive tumor cells). The final quantification of each staining was obtained by multiplying these 2 scores. A total staining score of 0–12 was calculated and graded as negative (−, score 0–1), weak (+ , score 2–4), moderate (+ + , score 5–8), or strong (+ +  + , score 9–12). We used ImageJ software for quantitative immunohistochemical analysis. All the use rights of patient specimens were approved by the Ethics Committee of the First Affiliated Hospital of The Army Medical University and obtained the informed consent of the patients.

### Lentiviral vectors construction and gene transfection

Lentiviral vectors were purchased and constructed by Genechem. The Interference sequences CARM1 was designed and synthesized by Shanghai Genechem. Target sequence of shRNA are listed as follow: Negative Control 5’TTCTCCGAACGTGTCACGT3', shRNA1 5’ctATGACTTGAGCAGTGTTAT3’, shRNA2 5’gcAGAACATGATGCAGGACTA3’. MM cell lines were plated in 12-well culture plates ($3 \times 10^5$ cells per well) containing RPMI-1640 medium. The lentivirus was successively added into the medium was fully mixed. After 16 h, the medium containing virus was replaced with fresh 10% FBS RPMI-1640 medium. Then, we observed the cell growth status after 72 h with an inverted fluorescence microscope.

### Real-time PCR

Total RNA was extracted by using RNAiso Plus (9108, Takara). Equal amounts of RNA were used for RT reaction according to manufacturer’s instructions (PrimeScript RT reagent Kit with gDNA Eraser, RR047A, Takara). Real-time quantitative PCR was performed with SYBR Green master Mix (208,054, Qiagen). GAPDH expression was used as control.

Primer sequence: CARM1 forward 5′AGCACCTACAACCTCAGCA3′, reverse 5′GGCTGTTGACTGCATAGTGG3′; GAPDH forward 5′CAATGACCCCTTCATTGACC3′, reverse5′GATCTCGCTCCTGGAAGATG3′; p53 forward 5′′GAGGTTGGCTCTGACTGTACC3′, reverse 5′TCCGTCCCAGTAGATTACCAC3′.

### Western blots

Western blots were utilized to measure the protein levels of CARM1 in MM cells. In brief, around 20 µg protein per sample was extracted and whole-cell lysate were electrophorezed by 10% SDS polyacrylamide gel electrophoresis (SDS-PAGE) and electrotransferred onto PVDF (polyvinylidene fluoride) membranes. Antigen and antibody complexes were detected with an ECL protocol using HRP-conjugated IgG as secondary antibodies.

### Cell proliferation and viability assay

Cell viability was assessed using the CCK-8 Cell Proliferation and Cytotoxicity Assay Kit following the manufacturer’s protocol (CA1210, Solarbio). In brief, MM cells were plated at 10,000 cells/ per well in a 96-well plate for 3 h. A multifunctional microplate reader (Thermo Varioskanfla, USA) was used to detect the OD values by each group of cells at a wavelength of 450 nm. The growth curves were drawn with the time and the corresponding OD values.

### Clone formation assay

Clonogenic formation was monitored by plating 10,000 MM cells in 300ul Methylcellulose Medium for Human cells (H4434, Stem Cell) with 2% FBS and 1% penicillin and streptomycin solution in 12-well plate. The cells were incubated at 37 °C with 5% CO_2_ for 1–2 week. The colonies were captured by Leica DMi8.

### Flow cytometry analysis of apoptosis and cell cycle

For apoptosis analysis, cells were stained with Annexin V APC for 15 min at 37 °C, and then add DAPI before analyzed by flow cytometry. For cell cycle analysis, was assessed using the EdU Alexa Fluor™ 647 Flow Cytometry Assay Kit following the manufacturer’s protocol. Flow cytometry equipped with BD FACSAria SORP. Experiments were performed in triplicate.

### RNA-seq and data analysis

Total RNA was extracted using Trizol reagent (Invitrogen, CA, USA) following the manufacturer's procedure. The total RNA quantity and purity were analyzed of Bioanalyzer 2100 and RNA 6000 Nano LabChip Kit (Agilent, CA, USA) with RIN number > 7.0. Approximately 10 ug of total RNA representing a specific adipose type was subjected to isolate Poly (A) mRNA with poly-T oligo attached magnetic beads (Invitrogen). Following purification, the mRNA is fragmented into small pieces using divalent cations under elevated temperatures. Then the cleaved RNA fragments were reversed-transcribed to create the final cDNA library following the protocol for the mRNA Seqsample preparation kit (Illumina, San Diego, USA), the average insert size for the paired-end libraries was 300 bp (± 50 bp). And then we performed the paired-end sequencing on an Illumina sequence platform.

### Immunofluorescent staining

The cells were fixed with 4% Paraformaldehyde, permeabilized with PBS containing 0.1% Triton X-100, and blocked with 2% BSA. After overnight incubation with primary antibodies (p53, 2527 T, Cell Signaling Technology) at 4 °C, the slides were incubated with corresponding secondary antibodies (Goat Anti-Rabbit, A32732, invitrogen). The images were captured by using a confocal microscope (ZEISS 880, Germany).

### Statistical analysis

Graph Pad Prism 8.0 was used for statistical analysis, and data were expressed as mean ± SD. Two-tailed Student’s t-test and one-way analysis of variance (ANOVA) (≥ three groups) were used to determine significance between experimental groups. The Kaplan–Meier method was used to evaluate the correlation of CARM1 expression with myeloma patient survival. In all cases, significance was defined as P < 0.05.

## Results

### Database and sample studies of patients with MM show that CARM1 expression is associated with MM prognosis

**Fig.1:**
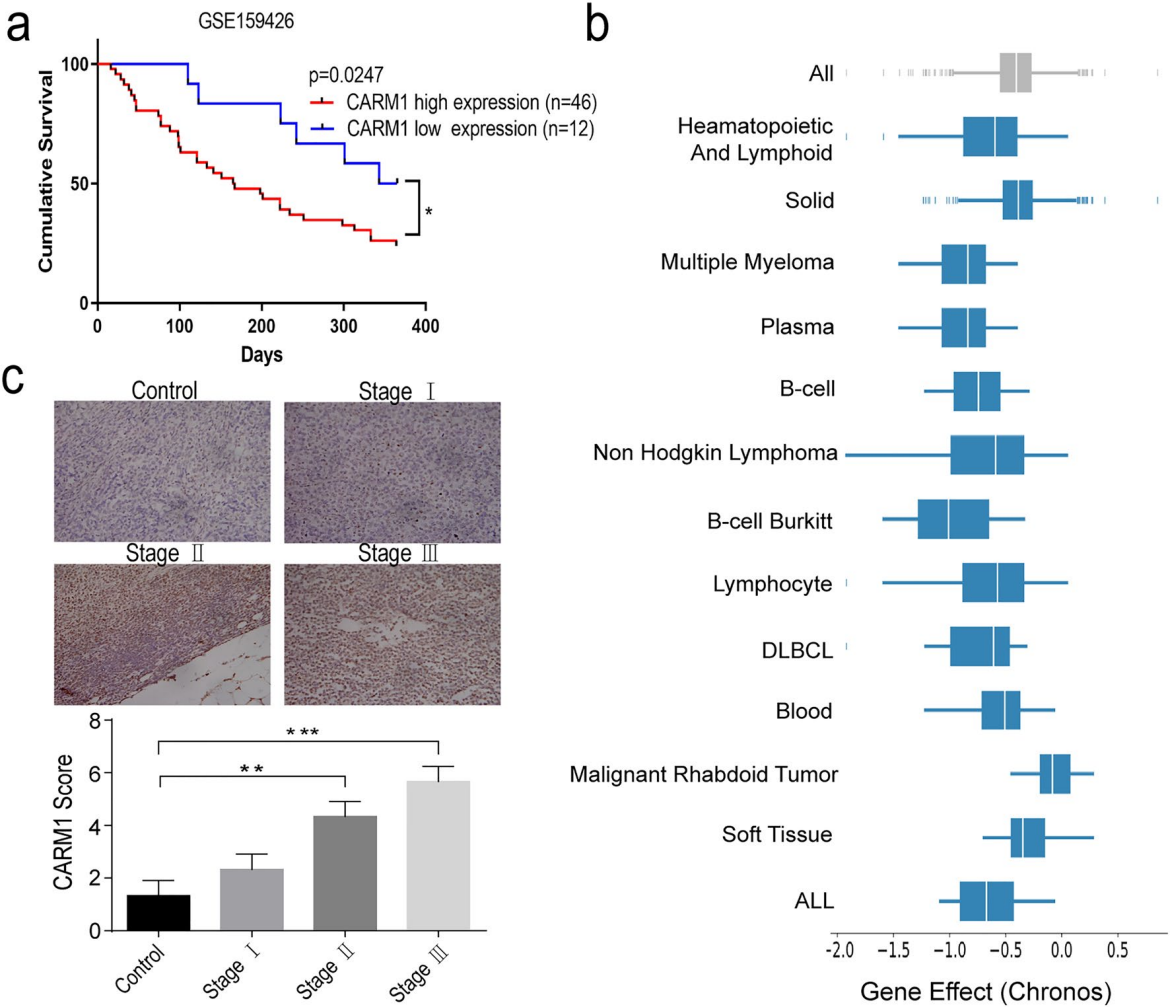
CARM1 serves as a poor prognostic biomarker in multiple myeloma. **a** High expression of CARM1 indicated poor overall survival (OS) in MM. **b** Cancer cell line dependency scores obtained from http://depmap.org. Haematopoietic And Lymphoid (1.3e-24) n = 118, Solid (1.3e-24) n = 968, Multiple Myeloma (2.2e-17) n = 21, Plasma Cell (2.2e-17) n = 21, B-cell (1.5e-12) n = 28, Non Hodgkin Lymphoma (5.9e-09) n = 28, B-cell Burkitt (3.8e-08) n = 6, Lymphocyte (2.0e-07) n = 37, DLBCL (5.0e-06) n = 9, Blood (6.7e-06) n = 60, Malignant Rhabdoid Tumor (8.9e-06) n = 10, Soft Tissue (2.4e-04) n = 48, ALL (2.4e-04) n = 17. **c** IHC staining verified that the CARM1 protein level was elevated in MM patient samples. (n = 4 each stage) Experiments were repeated three times independently. All data are displayed as mean ± SD; *P < 0.05, **P < 0.01, ***P < 0.001

To explore the role of CARM1 in MM, we analyzed The Cancer Genome Atlas MM dataset to determine whether CARM1 expression correlates with prognosis. Indeed, CARM1 high-expressing patients had significantly worse survival outcomes than patients with low CARM1 expression (p = 0.0247) (Fig. 1a). CARM1 is over-expressed in multiple cancer cell lines and patient samples. In addition, analysis from the DepMap website using CRISPR-Cas9 technology to screen for the necessity of various genes for tumors found that multiple myeloma, hematopoietic cells, and lymphocytes appear to be particularly dependent on CARM1 (Multiple Myeloma (2.2e-17) n = 21, Haematopoietic And Lymphoid (1.3e-24) n = 118, Lymphocyte (2.0e-07) n = 37) (Fig. 1b). We further detected CARM1 protein expression in MM patients’ bone marrow tissues compared with normal controls by IHC. Consistently, CARM1 was highly expressed in MM patients, and the expression level of CARM1 was significantly higher in stage III patients than in stage I-II patients (P < 0.05) (Fig. 1c), indicated that CARM1 is proportional to the severity of MM. In summary, CARM1 expression was elevated in MM patients and correlated with a worse prognosis.

### Expression of CARM1 in MM cell lines and its knockdown cell line constructed

**Fig. 2:**
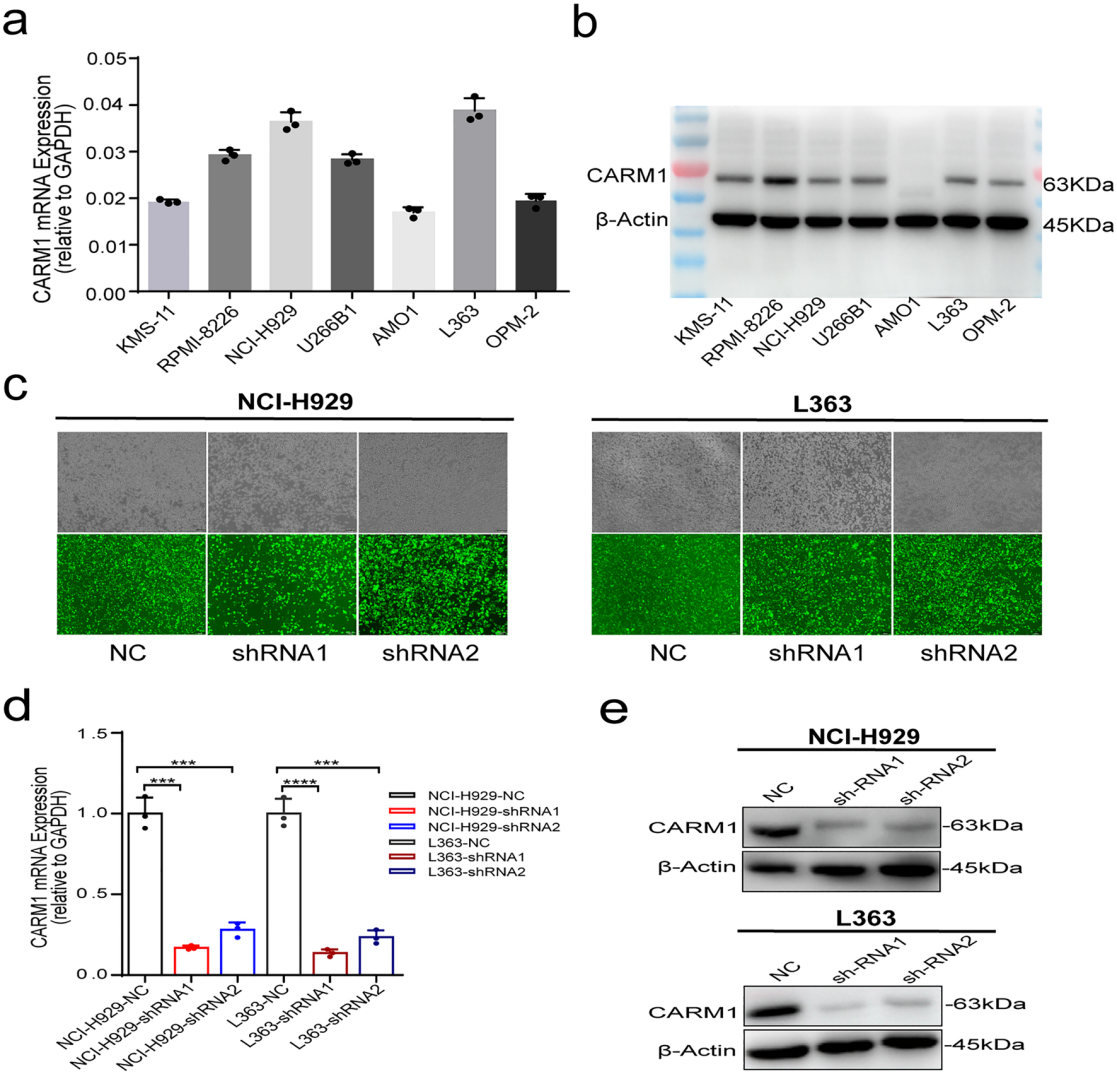
Screening and identification of CARM1 Knockdown cell lines in MM. **a** RT-qPCR detection of CARM1 mRNA expression in MM cell lines. **b** Western Blot detection of CARM1 protein expression in MM cell lines. **c** NCI-H929 cells and L363 cells transfected with CARM1-KD shRNA lentivirus under a fluorescence microscope. **d** RT-qPCR detection of CARM1 mRNA expression in NCI-H929 cells and L363 cells before and after two different small hairpin RNAs (shRNAs) transfection. **e** Western Blot detection of CARM1 protein expression in NCI-H929 cells and L363 cells before and after two different small hairpin RNAs (shRNAs) transfection. β-Actin was used as a loading control. Experiments were repeated three times independently. All data are displayed as mean ± SD; ***P < 0.001, ****P < 0.0001

To understand the biological function of CARM1 in MM cells, we detected the mRNA (Fig. 2a) and protein (Fig. 2b) expression of CARM1 in 7 MM cell lines. We found that the mRNA and protein expression of CARM1 were not consistent in cells. We speculated that CARM1 not only depends on the regulation of transcriptional activity but is also determined by post-transcriptional regulation. To better explore the role of CARM1 in MM cells, we selected NCI-H929 and L363 cell lines with both high mRNA and protein levels of CARM1 for follow-up research. To evaluate the biological basis of CARM1 dependency, we generated vectors that express two different small hairpin RNAs (shRNAs) that efficiently target CARM1 (Fig. 2c). We examined the effects of CARM1 knockdown on NCI-H929 cells and L363 cells. The MM cell lines transduced with these vectors showed significantly decreased CARM1 mRNA and protein expression (Fig. 2d and e).

### Knockdown of CARM1 leads to cell cycle arrest and induces apoptosis in MM cell lines.

**Fig. 3:**
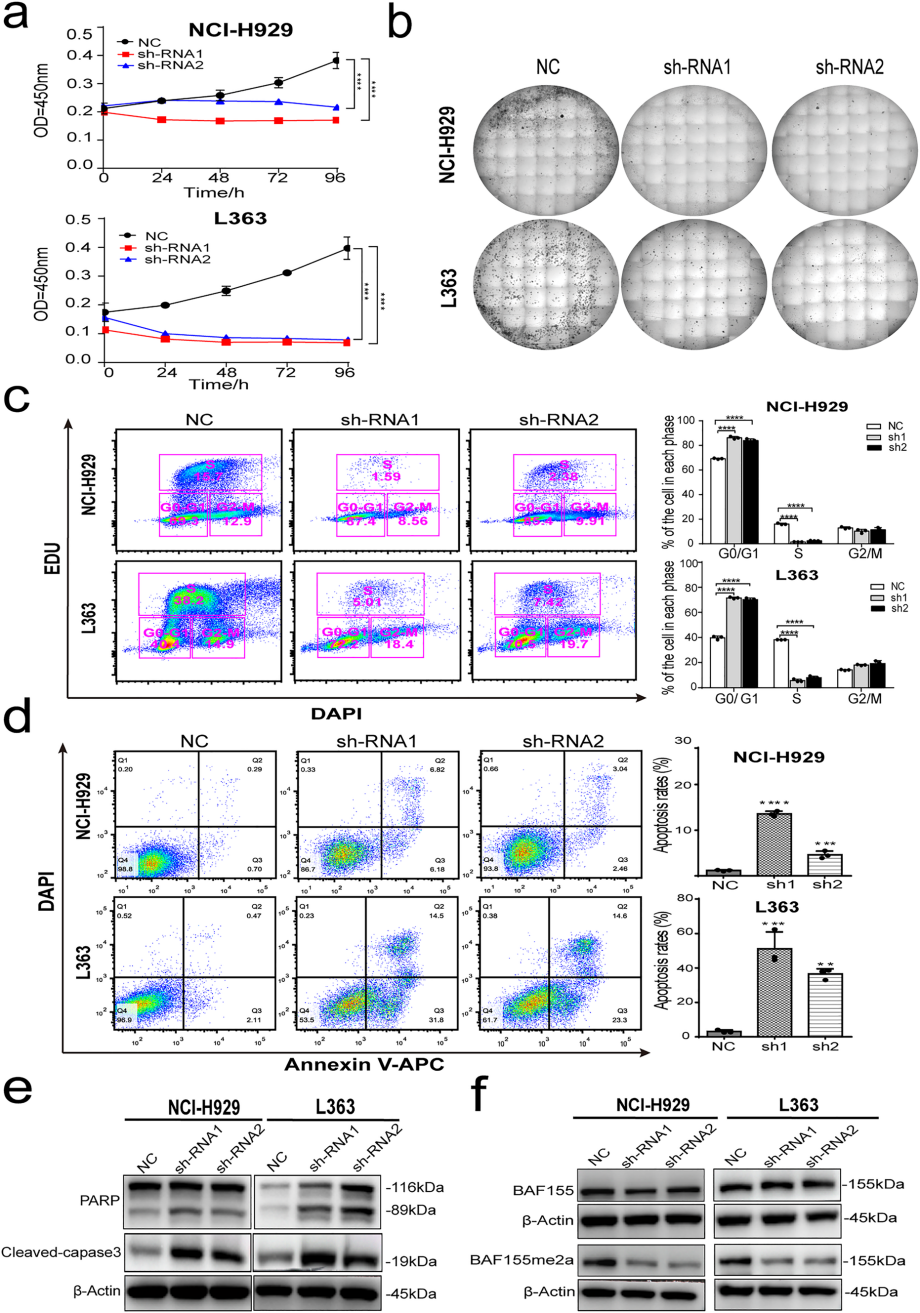
CARM1 knockdown induces growth inhibition and apoptosis in multiple myeloma cell lines. **a** CCK-8 cell proliferation assay on CARM1-KD NCI-H929 and L363 cells compared with negative control cells (NC). **b** Clonogenicity evaluation for the NC and CARM1-KD NCI-H929 and L363 cells. **c** Flow cytometry for cell cycle marker EDU in MM cells after CARM1-shRNA lentivirus transfection for 72 h. **d** Flow cytometry for cellular apoptosis marker Annexin V in MM cells after CARM1-shRNA lentivirus transfection for 72 h. **e** Western Blot detection of apoptosis-related proteins cleaved-caspase-3 and PARP expression in NCI-H929 cells and L363 cells before and after two different small hairpin RNAs (shRNAs) transfection. β-Actin was used as a loading control. **f** Western Blot detection of BAF155 dimethylation protein expression in NCI-H929 cells and L363 cells before and after two different small hairpin RNAs (shRNAs) transfection. β-Actin was used as a loading control. Experiments were repeated three times independently. All data are displayed as mean ± SD; **P < 0.01, ***P < 0.001, ****P < 0.0001

To expose the effect of CARM1 on MM cell growth, we assessed the cell viability of NCI-H929 KD cells and L363 KD cells at 0 h, 24 h, 48 h, 72 h, and 96 h using a CCK-8 kit (Fig. 3a). The analysis showed that cellular viability was significantly inhibited by CARM1 knockdown compared with negative control, suggesting that CARM1 boosted MM cell proliferation in vitro. The growth inhibition effect of CARM1 knockdown was further confirmed by a clone formation assay. As shown in Fig. 3b, the Knockdown of CARM1 reduced cloning capability compared with control cells. Next, we examined cell-cycle progression and observed that the knockdown of CARM1 significantly decreased the cell proportion of the S phase and induced G0/G1 arrest for NCI-H929 and L363 cell lines (Fig. 3c). Therefore, we evaluated whether cell cycle arrest induces apoptosis by CARM1 knockdown in MM cells. The flow cytometry showed that Annexin V positive cells significantly increased after CARM1-shRNA transfection for 72 h (Fig. 3d). Additionally, CARM1 knockdown upregulated the expression of cleaved PARP and cleaved-capase-3 in MM cells (Fig. 3e). Cleavage of PARP promotes cell disassembly and is widely used as a marker of apoptosis [18, 19]. Caspase-3 is a key executor of apoptosis and is mainly responsible for PARP cleavage [20, 21]. As expected, western blotting analysis confirmed that CARM1 knockdown promoted apoptotic protein expression in MM cells. Furthermore, To confirm that shRNA affects methyl transfer, we evaluated the dimethylation level of the BRG1-associated factor (BAF155). BAF155 is the direct substrate of CARM1; arginine dimethylation of BAF155 is significantly reduced in CARM1-deficient cells [22]. Consistent with literature reports, western blotting assays indicated that CARM1-KD decreased the dimethylation of BAF155 in MM cells (Fig. 2f). In conclusion, the decreased growth rate of CARM1-KD cells was ascribed to cell cycle arrest which induced apoptosis by CARM1 knockdown.

## .

### Transcriptional analysis of CARM1 knockdown in MM cell lines

**Fig. 4:**
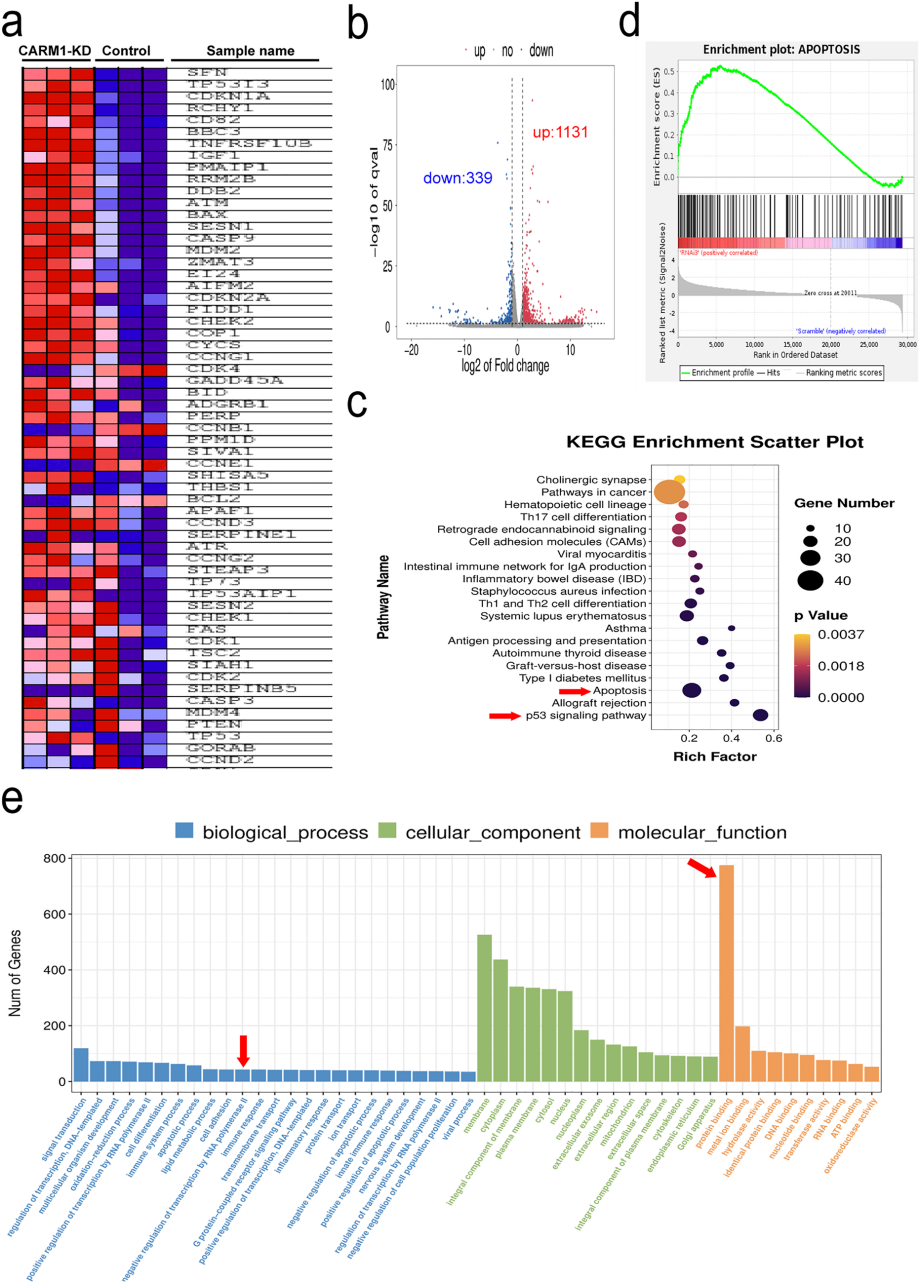
Transcriptional Analysis of CARM1 Knockdown in MM Cell Lines. **a** Heat maps showed the top 40 different genes in the constructed cells. **b** The Volcano plot shows the genes that are up-regulated (red) and down-regulated (blue) in expression after CARM1 knockdown. **c** Pathway enrichment analysis of KEGG revealed that CARM1 was correlated to apoptosis and the p53 signaling pathway. **d** Representative GSEA plot depicting the upregulation of apoptosis. **e** GO analysis of RNA-seq

**Fig. 5:**
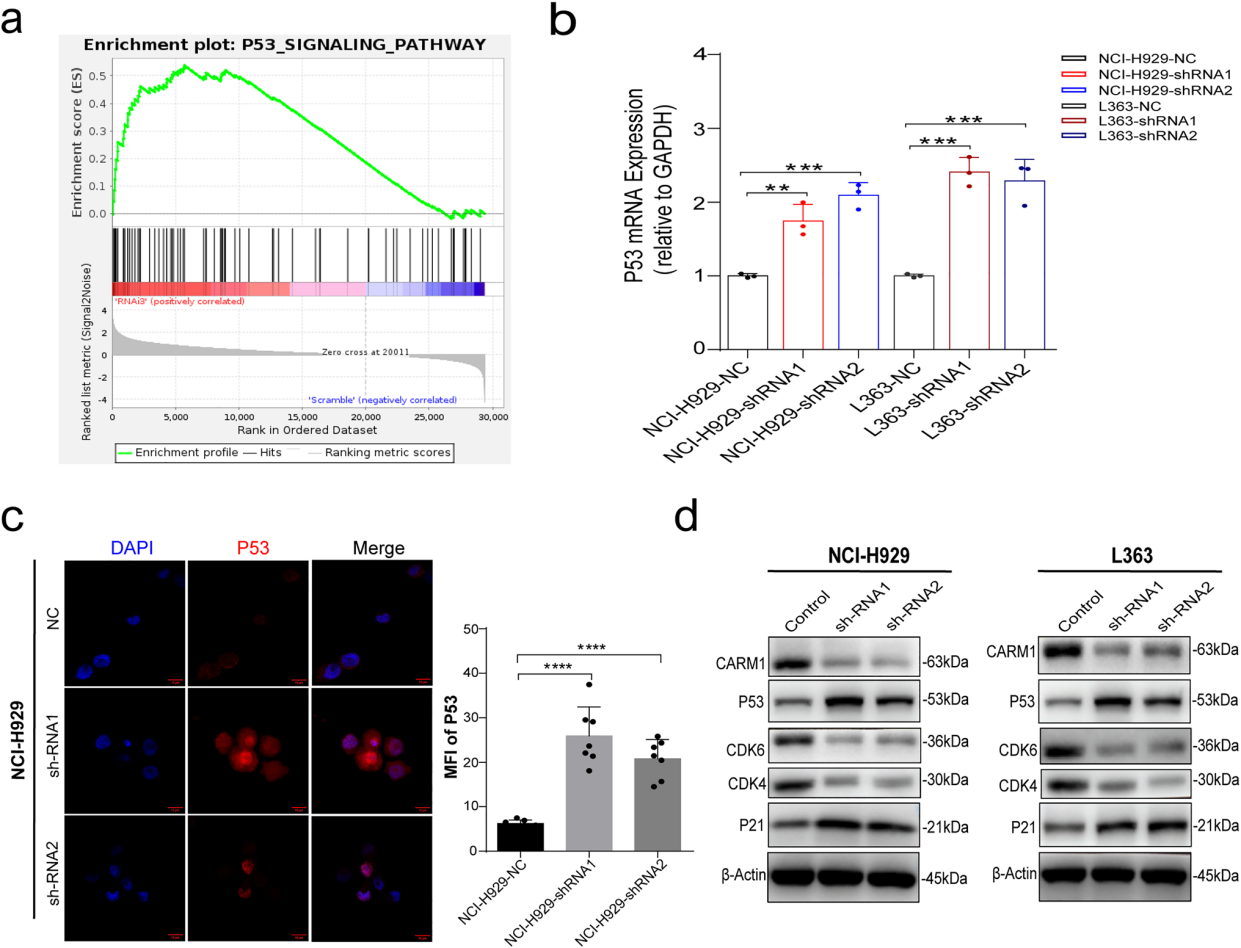
Targeting CARM1 inhibits the progression of MM by activating the p53 pathway. **a** GSEA enrichment analysis showed that the p53 pathway was significantly upregulated. **b** RT-qPCR results showed that mRNA expression of p53 was significantly elevated after CARM1 knockdown in NCI-H929 cells and L363 cells. **c** Immunofluorescence staining on NCI-H929 after CARM1-shRNA lentivirus transfection for 72 h. DAPI (blue); p53 (red). Scale bar, 20 µm. The graph shows the average fluorescence intensity of p53-positive punctate cells (right). **d** Western blotting analysis confirmed that MM cells after CARM1 knockdown were extracted and proteins were incubated with primary antibodies against CARM1, CDK4, CDK6, p53, and p21. β-Actin was used as a loading control. Experiments were repeated three times independently. All data are displayed as mean ± SD; **P < 0.01, ***P < 0.001, ****P < 0.0001

To define the mechanism of CARM1 in MM cells, we performed gene expression analyses by RNA sequencing (RNA-seq) in NCI-H929 after CARM1 knockdown. The heat map showed the top 56 differential genes based on the p value < 0.01, the altered genes including p53, p16, p21, etc. cell cycle block genes, and bax (apoptosis-related gene) increased their expression, while the expression of CCNB1, CDK4, etc. cell cycle-related and Bcl-2 (apoptosis resistance) genes was reduced after CARM1 knockdown (Fig. 4A). We used the volcano map to show the differential gene expression affected by CARM1 knockdown. A total of 1470 genes were affected by CARM1 knockdown in the NCI-H929 cell line, of which 1131 genes were up-regulated and 339 genes down-regulated (Fig. 4B). Then, we used KEGG enrichment analysis in CARM1 knockdown cells and controls (Fig. 4C), which revealed that after CARM1-knockdown significantly enriched in apoptotic pathways. Gene set enrichment analysis (GSEA) also showed that apoptosis and p53 signaling pathways were enriched after CARM1 knockdown (Figs. 4D and 5A). In addition, Fig. 4E shows that CARM1 can negative regulation of transcription by RNA polymerase II and protein binding, indicating that CARM1 might negatively regulate the transcription of p53 by binding transcription factors.

### Knockdown of CARM1 activates p53 signaling pathway in MM cell lines

To define the molecular pathways regulated by CARM1, we also applied GSEA to identify the most alternative pathways influenced by the loss of CARM1 and found that the p53 signaling pathway was significant up-regulation (Fig. 5a). Furthermore, we found that the expression of p53 mRNA was significantly up-regulated by CARM1 KD in NCI-H929 cells and L363 cells (Fig. 5b). Meanwhile, immunofluorescence staining confirmed that the protein level of p53 significantly increased by CARM1 KD compared to NC cells (Fig. 5c), suggesting that the p53 pathway was activated. To better understand the mechanisms leading to the cell-cycle arrest and induction of apoptosis by CARM1 KD in MM cells, we next analyzed changes in selected proteins by western blotting. The results showed that the level of p53 was elevated and expression of p21 was also stimulated (Fig. 5d). In addition, we tested whether CARM1 could affect the expression of CDK4 and CDK6 in MM cells. CDK4/6 are key initiators of the G1-to-S phase transition, while inhibition of CDK4/6 leads to G1 arrest of the cell cycle [23]. The results showed that the expressions of CDK4 and CDK6 were decreased by CARM1 KD (Fig. 5d). In conclusion, we considered that inhibition of CARM1 will activate the p53 signaling pathway and induce cell cycle arrest and apoptosis of MM cells.

### Knockdown of CARM1 sensitizes MM cell lines chemotherapeutic of bortezomib

**Fig. 6:**
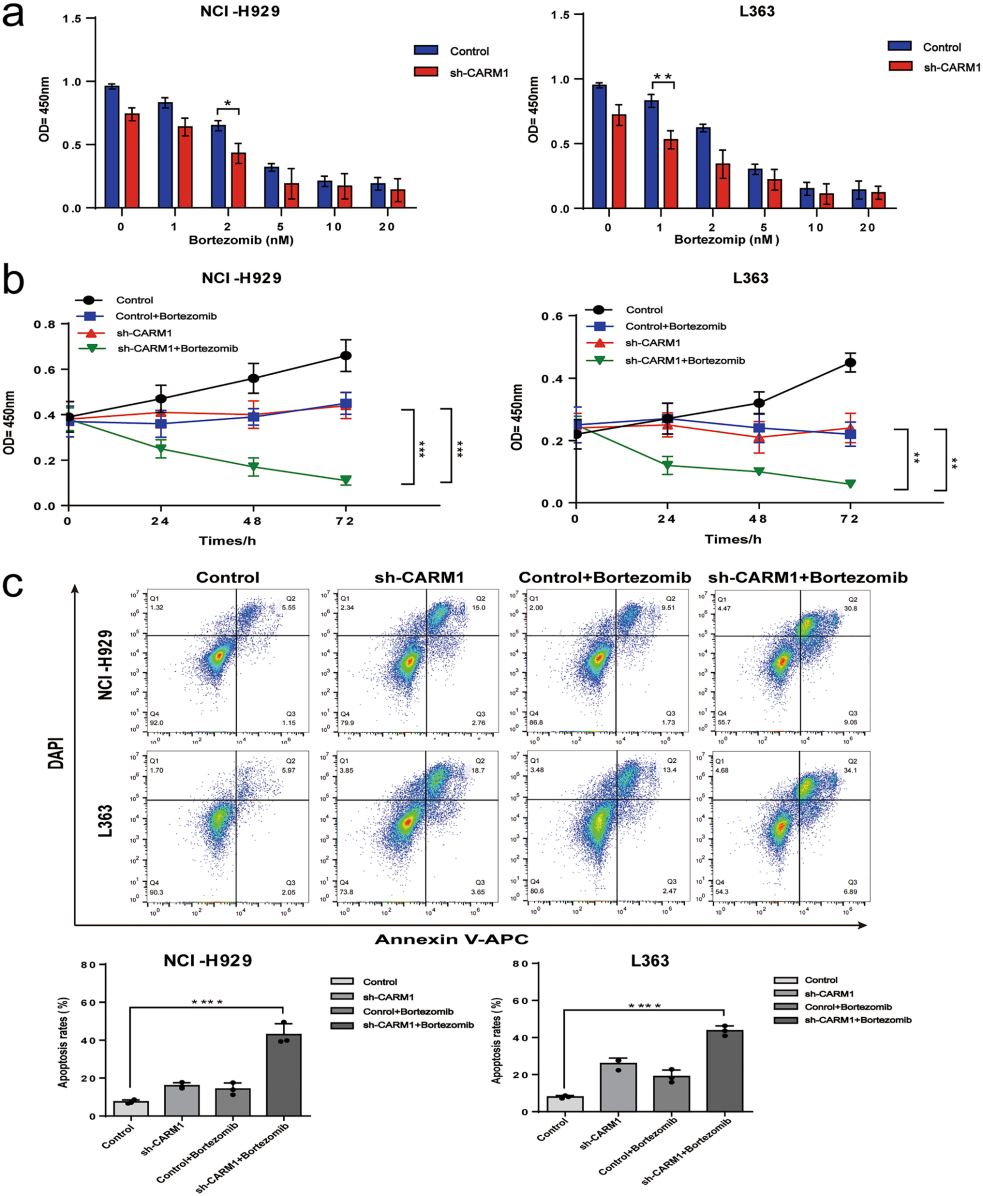
Knockdown of CARM1 enhances the therapeutic effect of bortezomib. **a** NCI-H929 and L363 cells were treated with various of bortezomib for 24 h and cell viability was measured by CCK-8 kit. **b** NCI-H929 and L363 cells were treated with 2 nm and 1 nm bortezomib respectively at different times and cell viability was measured by CCK-8 kit. **c** The stable CARM1 knockdown cell lines (NCI-H929 and L363) and controls were treated with 2 nm and 1 nm bortezomib respectively for 24 h and the percentage of apoptosis cells was measured using flow cytometry. Experiments were repeated three times independently. All data indicate the mean ± SD.*p < 0.05, **p < 0.01, ***p < 0.001, ****p < 0.0001

Almost all patients with multiple myeloma eventually relapse.The median progression-free survival (PFS) and overall survival (OS) in patients with relapsed multiple myeloma refractory to lenalidomide and bortezomib is poor, with median times of 5 months and 9 months, respectively [24]. We treated NCI-H929 and L363 and their stable CARM1 knockdown cell lines with different doses of bortezomib (0 nm, 1 nm, 2 nm, 5 nm, 10 nm, 20 nm) for 24 h and detected the cell viability (Fig. 6a), which demonstrated the IC50 of NCI-H929 and L363 CARM1 knockdown cell lines is 2 nm and 1 nm, respectively. There was a significant difference in cell viability at the same drug concentration between the control group and PC4 knockdown cell lines. Then, NCI-H929 and L363 constructed cells were treated with 2 nm and 1 nm bortezomib respectively to detect cell viability at different time points (0 h, 24 h, 48 h, 72 h), which showed that the inhibition effect of CARM1 knockdown combined with bortezomib treatment on cell viability was better than that of CARM1 knockdown alone or bortezomib treatment alone (Fig. 6b). As shown in Fig. 6C, the knockdown of CARM1 combined with bortezomib treatment significantly increased apoptosis compared with CARM1 knockdown alone or bortezomib treatment alone. The above data demonstrated that the Knockdown of CARM1 could enhance the chemosensitivity of bortezomib in MM cell lines.

## Discussion

Multiple myeloma (MM) remains an incurable hematologic malignancy with a high proliferative profile, especially in high-risk subgroups of patients [25], and although the development of new proteasome inhibitors and other therapeutic strategies has greatly improved the survival of MM patients, acquired drug resistance and malignant proliferation ultimately lead to relapse and poor prognosis [26], and the exact mechanisms remain incompletely elucidated. In addition, the prognostic stratification and therapeutic assessment system for MM lacks specific molecular indicators. Therefore, the search for new biomarkers and potential therapeutic targets, especially for relapsed refractory MM, is imminent.

CARM1, an important target in cancer, is closely related to tumor cell proliferation. CARM1-KD promotes the proliferation of lung cancer cells [27], and the down-regulation of CARM1 significantly inhibits the proliferation of gastric cancer cells [28]. In contrast, the proliferation role of CARM1 in MM and the mechanism of its occurrence have been rarely reported. Studies have reported that the use of selective inhibitors of CARM1 inhibits the proliferation of multiple myeloma cell lines and that affected cells are blocked in the G1 phase [16], which is consistent with our findings using shRNA to knock down CARM1 expression in MM cells. CARM1 is also an arginine methyltransferase, and in addition to the role of histone H3 methylation in the transcriptional activation of estrogen receptor (ER) target genes [29], methylation of non-histone substrates regulates different features of cancer [30], for example, BAF155 methylation drives cancer metastasis [31]. But it is not clear how CARM1 plays a role in MM.

In this study, we found that when the CARM1 gene was knockdown in MM cell lines, flow cytometry results showed increased apoptosis and induced cellular G0/G1 phase arrest. These two knockdown sequences differ in their apoptotic effects, and the knockdown sequences were validated on NCBI and found to belong to CARM1 sequences only. Therefore, we speculate that different sequences of CARM1 have different functions and lead to different apoptotic effects. This is the part that we will explore in the next study. Also, RNA-Seq analysis showed increased expression of cell cycle blocking genes and pro-apoptotic related genes, and apoptosis and p53 pathways were significantly enriched. The expression of CARM1 was also negatively correlated with p53 expression as verified by RT-qPCR and cellular immunofluorescence assays. In summary, our study showed that CARM1 knockdown induced G0/ G1 phase block and p53 pathway activation inhibited MM cell proliferation, and promoted apoptosis. In addition, there have been no reported that an association between CARM1 and p53 in MM, suggesting its specificity.p53 is one of the major determinants of the anti-proliferative response, integrating multiple stress signals to prevent abnormal cell growth and tumorigenesis [32]. It has been shown that CARM1 can interact with p53 and thus participate in regulating the methylation status of p53 target gene proteins [33, 34]. Another study found that CARM1 can regulate the expression of p53 target genes [35]. In our study, we found that knockdown of CARM1 in MM cell lines affected the expression of p53 at the mRNA and protein levels, while a significant decrease in the dimethylation level of BAF155 (the direct substrate of CARM1) by Western blotting assay, indicating that our constructed vectors expressing two different small hairpin RNAs (shRNAs) effectively targeted CARM1 and affected methyl transfer. Therefore, we speculate that CARM1 may directly inhibit the transcription of p53 through transcriptional repression, while CARM1 may also have an indirect effect on p53 protein methylation.

Although the experimental results obtained in this study were more than satisfactory, there are still shortcomings. The use of CARM1 inhibitors in this study was only studied on cells, and in subsequent studies cellular experiments with Crispr-cas9 should be continued in depth and the effects of inhibitors on hormonal experiments should be completed on animals. In addition, We have not yet conducted animal experiments to investigate whether the effects of CARM1 in MM in vivo and its effects are consistent with those in vitro, and we will need to supplement the validation in the future. Certainly, our current understanding of the role of CARM1 in regulating proliferation and apoptosis in multiple myeloma through the p53 pathway is just beginning, and the related molecular mechanisms still need to be explored and studied in greater depth.

## Conclusions

This study shows that high expression of CARM1 in multiple myeloma patients, especially in stage III or relapsed refractory MM, is strongly associated with poor prognosis; CARM1 knockdown activates the p53 signaling pathway and inhibits MM cell proliferation. In addition, combining shRNAs that effectively target CARM1 with bortezomib showed significant inhibitory effects on MM cells. This study on CARM1 is of great significance in elucidating the pathogenesis of MM and finding potential therapeutic targets.

## Abbreviations

CARM1: The enzyme co-activator associated arginine methyltransferase 1
MM: Multiple myeloma
BM: Bone marrow
FBS: Fetal bovine serum
IHC: Immunohistochemistry
MFI: Mean fluorescence intensity
